# Real-world depression, anxiety and safety outcomes of intramuscular ketamine treatment: a retrospective descriptive cohort study

**DOI:** 10.1186/s12888-022-04268-5

**Published:** 2022-10-03

**Authors:** Sachin Ahuja, Madeline Brendle, Leo Smart, Claire Moore, Paul Thielking, Reid Robison

**Affiliations:** 1 Cedar Psychiatry, Springville, UT USA; 2 Department of Pharmacotherapy, University of Utah College of Pharmacy, Salt Lake City, UT USA; 3 Numinus Wellness, Vancouver, British Columbia Canada; 4 University of Utah School of Medicine, DraperSalt Lake City, UT 721 E 12200 S, 84020 USA

**Keywords:** Ketamine, Intramuscular ketamine, Real-world data, Depression, Suicidal ideation, Anxiety, Safety

## Abstract

**Background:**

Ketamine has emerged as a promising pharmacotherapy for depression and other mental illnesses, and the intramuscular (IM) administration of ketamine is now offered at many North American outpatient psychiatric clinics. However, a characterization of the outpatient population receiving IM ketamine treatment and an evaluation of the real-world depression, anxiety, and safety outcomes of long-term psychiatric IM ketamine treatment has not been reported. This study aimed to evaluate the clinical characteristics, treatment patterns, clinical outcomes, and adverse events of patients receiving IM ketamine treatment.

**Methods:**

Patient data from the electronic health records of a private outpatient psychiatric clinic network in the United States were collected and analyzed retrospectively. Adults with any psychiatric diagnosis who received ketamine treatment only by IM administration from January 2018 to June 2021 were included. A total of 452 patients were included in the cohort.

**Results:**

Patients receiving IM ketamine treatment had a mean of 2.8 (SD 1.4) psychiatric diagnoses. 420 (93%) patients had a diagnosis of major depressive disorder, 243 (54%) patients had a diagnosis of generalized anxiety disorder, and 126 (28%) patients had a diagnosis of post-traumatic stress disorder. Patients received a median of 4 (range 1–48) IM ketamine treatments. Median depression scores (PHQ-9) improved 38% from 16.0 (IQR 11.3–21.8) at baseline to 10.0 (IQR 6.0–15.0) at last treatment (*p* < .001). Median anxiety scores (GAD-7) improved 50% from 14.0 (IQR 8.0–17.0) at baseline to 7.0 (IQR 4.3–11.8) at last treatment (*p* < .001). With maintenance ketamine treatments, average improvements in depression (PHQ-9) and anxiety (GAD-7) scores of at least 4.7 and 4.9 points were maintained for over 7 months. An adverse event occurred during 59 of 2532 treatments (2.3%).

**Conclusions:**

IM ketamine is being utilized to treat psychiatric outpatients with multiple mental illnesses not limited to depression. Average depression and anxiety levels significantly improve throughout IM ketamine treatment and do not regress to baseline during patients’ maintenance treatment phase. Prospective studies are recommended to confirm the long-term effectiveness and safety of IM ketamine.

**Supplementary Information:**

The online version contains supplementary material available at 10.1186/s12888-022-04268-5.

## Background

Ketamine is a glutamatergic n-methyl-_D_-aspartate receptor antagonist that was originally approved by the United States (US) Food and Drug Administration (FDA) in 1970 as a general anesthetic [1]. Over the past two decades, subanesthetic ketamine has emerged as a psychopharmacotherapy option for major depressive disorder (MDD) due to its rapid antidepressant and antisuicidal effects [2–5]. Ketamine treatment has also demonstrated efficacy for patients with other mental illnesses besides MDD. It has been shown to reduce anxiety symptoms in patients with generalized anxiety disorder (GAD) and/or social anxiety disorder (SAD) [6–8], and preliminary evidence shows that ketamine treatment may reduce symptoms of substance use disorders [9–12], post-traumatic stress disorder (PTSD) [13], bipolar depression [14], and eating disorders [15].

Ketamine is a racemic mixture of two enantiomers: (R)-ketamine and (S)-ketamine (esketamine) [1]. Esketamine has been approved by the FDA for adults with treatment-resistant depression (TRD) or MDD with acute suicidal ideation or behaviour [16, 17]. However, in many countries, physicians are also able to prescribe racemic ketamine for off-label psychiatric indications. Racemic ketamine can be administered via intravenous (IV), intranasal (IN), oral, sublingual (SL), anal, subcutaneous, and intramuscular (IM) routes [18]. Repeated-dose IV ketamine has demonstrated efficacy for the treatment of MDD [19–21], and IV ketamine has become a commonly offered off-label treatment for MDD at ketamine infusion clinics across North America and Europe [22].

While the majority of research on racemic ketamine treatment has been on IV ketamine, initial studies show that racemic IM ketamine treatments show similar mental health outcomes to IV ketamine infusions. In a 2014 study in India, 18 patients with treatment-refractory MDD who received either 0.5 mg/kg IM ketamine or 0.5 mg/kg IV ketamine showed a similar 60 and 59% reduction in depressive symptoms at 2 h after injection (Hamilton Depression Rating Scale) [23]. Furthermore, in a case series of 40 MDD patients who received six IM ketamine treatments over 3 weeks, patients showed reductions in depression (Patient Health Questionnaire, PHQ-9), anxiety (Generalized Anxiety Disorder-7, GAD-7) and PTSD (PTSD Checklist for DSM-V) symptoms of 55, 51 and 51% respectively [24], which is analogous to IV administration [21].

While IM ketamine has initially showed similar efficacy to IV ketamine, it may also be a more convenient and practical option for mental health treatment in community psychiatric clinics and primary care clinics [23]. This is because IM administration does not require the use and monitoring of an IV infusion pump [23, 25], and it is relatively less expensive than IV administration [23, 24]. IM ketamine treatment is currently being offered by specialized psychiatric clinics in North America as an off-label indication to treat a variety of mental illnesses [26–31].

Nonetheless, real-world evidence on psychiatric IM ketamine treatment are limited. One real-world study has described the population of patients receiving a similar treatment: Dore et al. reported patient demographic, diagnosis, and outcome data for patients receiving IM and SL ketamine-assisted psychotherapy from three private general psychiatric practices [18]. Aside from the study by Dore et al., the population of outpatients receiving psychiatric IM ketamine treatment has not been characterized, and the treatment protocols used by the various clinics offering this medication have not been reported. Furthermore, real-world evidence on mental health outcomes for patients who receive IM ketamine treatment is limited. Two real-world studies have shown that patients with MDD show significant improvements in depression and anxiety symptoms after a series of six to eight IM ketamine treatments [24, 32]. However, depression and anxiety outcomes associated with long-term maintenance IM ketamine treatments have not been reported. Finally, evidence on the adverse event (AE) profile of outpatient psychiatric IM ketamine treatment has not been reported.

Given the limited data and potential for IM ketamine as an effective treatment option for patients with MDD and other mental illnesses, there is a need to evaluate patients receiving IM ketamine at outpatient psychiatric clinics. In this retrospective descriptive cohort study, we describe the clinical characteristics, treatment patterns, clinical outcomes and AEs of outpatients who received psychiatric IM ketamine treatment.

## Methods

This retrospective descriptive cohort study was conducted in accordance with International Society for Pharmacoepidemiology Guidelines for Good Pharmacoepidemiology Practices and received ethics clearance from the University of Utah Institutional Review Board.

The inclusion criteria were patients 18 years or older who received IM ketamine treatment at the private outpatient psychiatric clinic network between January 1st, 2018 and June 30th, 2021. Patients who received IV, oral, or SL ketamine, or IN esketamine from the clinics during the study period were excluded from the study.

Patients received standard of care IM ketamine treatment at the psychiatric clinic. A clinician – either a psychiatrist or physician assistant – was responsible for each patient’s initial recommendation and prescription for ketamine treatment, and the ongoing management of their ketamine treatment plan including patients’ ketamine dosage, treatment frequency and number of ketamine treatments. During ketamine treatments, each patient was monitored using blood pressure, pulse, and direct observation for at least 60 minutes after the administration of ketamine by the prescribing clinician, with support from a medical assistant. The prescribing clinician was responsible for responding to any AEs that arose and recording the presence of AEs in the appropriate clinical notes. Patients received ketamine while laying in a reclining chair in a quiet room with dim lights. During the treatments, patients wore eye shades and listened to music through headphones. Patients selected the music from a collection of playlists consisting of relaxing non-lyrical music curated by clinic staff.

Study data was collected retrospectively from the psychiatric clinic’s electronic health records (EHR) system. Data was collected from the EHR through customizable reports and a medical chart review. The variables which were collected through customizable reports include patient demographics, self-reported medical, social and family history, treatment dates, and depression, suicidal ideation (SI) and anxiety outcomes. Some variables were not available as structured reports, so they were collected through a manual chart review. These variables include patients’ diagnoses, baseline concomitant medications, adverse events, vital signs, and ketamine dosage.

As part of routine clinical care, the Patient Health Questionnaire (PHQ-9) and Generalized Anxiety Disorder (GAD-7) surveys were self-administered by patients before each ketamine treatment session. The PHQ-9 and GAD-7 are valid and reliable measures to screen for MDD and GAD in clinical practice and research [33, 34]. The PHQ-9 asks patients to rate the frequency of the Diagnostic and Statistical Manual of Mental Disorders, 5th Edition’s (DSM-V) symptoms of MDD over the past 2 weeks [33]. The PHQ-9 total score ranges from 0 to 27, with scores representing minimal depression (0–4), mild depression (5–9), moderate depression (10–14), moderately severe depression (15–19), and severe depression (20–27). Item 9 of the PHQ-9 is a brief SI measure. This SI measure is scored on a scale of 0 to 3, and shows validity for use as a screening tool for suicide risk [35]. The GAD-7 prompts patients to rate the frequency of the DSM-V symptoms of GAD over the past 2 weeks [34]. The GAD-7 total score ranges from 0 to 21, and scores represent mild anxiety (0–5), moderate anxiety (6–10), moderately severe anxiety (11–15), and severe anxiety (15–21). The primary outcomes for the study were changes in the cohort’s median PHQ-9 and GAD-7 scores from patients’ first to last ketamine treatment.

### Statistical analysis

Descriptive statistics were used to summarize study variables. Some variables have missing data. History questionnaires were provided to patients to complete before their first visit; however, not all patients completed these questionnaires. Additionally, not all patients completed the PHQ-9 or GAD-7 before each ketamine treatment. The *N* with data for each variable is shown, and descriptive statistics are calculated based on the number of patients with data available.

Wilcoxon signed-rank tests were used to assess the change in PHQ-9 and GAD-7 scores from patients’ first to last ketamine treatment. Spearman’s rank correlations were used to evaluate the association between ketamine dose and patient weight as well as the association between the number of ketamine treatments patients received and their PHQ-9, SI and GAD-7 scores. STATA version 16 and IBM SPSS version 28 were used for statistical analyses. Levels of significance in this study were defined as *p* < .05.

## Results

A total of 452 patients were included in the study. 230 (51%) patients were female and patients’ median age at baseline was 36.4 (IQR 27.3–47.7) years. Additionally, 95% (365/384) of patients were white, non-Hispanic, and non-Latinx. For patients 25 years or older, 86% (179/207) had engaged in some post-secondary education (Table 1).

**Table 1:** Demographic characteristics and self-reported social and mental health history of patients receiving IM ketamine therapy

	No. of patients with available data	N (%)
**Demographic characteristics**		
Sex, female	452	230 (51%)
Age group	452	
18–29 years		155 (34%)
30–39 years		117 (26%)
40–49 years		78 (17%)
50–59 years		62 (14%)
≥ 60 years		40 (9%)
Race	410	
American Indian or Alaska Native		2 (1%)
Asian		7 (2%)
Black or African American		3 (1%)
Native Hawaiian or Other Pacific Islander		1 (0.2%)
White		397 (97%)
Ethnicity	395	
Hispanic or Latinx		16 (4%)
Utah residence	419	399 (95%)
Highest education level (25 years or older)	207	
10th/11th grade		3 (1%)
High school graduate or GED		25 (12%)
Some college (in progress or incomplete)		70 (34%)
Undergraduate degree		79 (38%)
Graduate degree		30 (14%)
Employed	276	181 (67%)
Living status	236	
Owns house/apartment		103 (44%)
Rents house/apartment		66 (28%)
Lives with parent(s)/family		55 (23%)
Lives with friend(s)/roommate(s)		12 (5%)
**Social History**		
History of physical or sexual abuse	210	99 (47%)
**Mental Health History**		
Previous psychotherapy	197	110 (56%)
Any self-harm behaviour	85	35 (41%)
Suicide attempts	114	42 (37%)
Psychiatric hospitalizations	199	61 (31%)
Attended a drug/alcohol treatment centre	233	29 (12%)

The self-reported social and mental health history of IM ketamine patients are summarized in Table 1. Of patients with available data, 47% (99/210) reported being a survivor of previous physical or sexual abuse, 37% (41/112) reported a history of self-harm behaviour, and 37% (42/114) reported a history of one or more suicide attempts. Additionally, 31% (61/199) of patients reported a history of having received inpatient treatment at a psychiatric hospital.

Regarding patients’ self-reported history of substance use at baseline (Supplemental Table 1), 2% (6/250) reported heavy alcohol consumption, 11% (32/281) reported being current tobacco smokers, 4% (10/233) reported current opiate use, and 20% (46/225) reported prior psychedelic drug use.

Of the 266 patients with completed self-report data on family mental health history (Supplemental Table 2), 236 patients (89%) had a family history of mental illness, and 37 patients (14%) had a family member who had attempted suicide. Also, 165 (62%) patients had a family history of depression, and 132 patients (50%) had a family history of an anxiety disorder.

All psychiatric diagnoses of patients are summarized in Table 2. All 452 patients had at least 1 psychiatric diagnosis, and patients had a mean of 2.8 (SD 1.4) psychiatric diagnoses. Approximately 420 patients (93%) had MDD and 437 patients (97%) had any mood disorder including MDD, bipolar disorder, dysthymic disorder, cyclothymic disorder, or unspecified mood disorder. Furthermore, 288 patients (64%) had an anxiety disorder which includes GAD, anxiety disorder unspecified, panic disorder, or phobic anxiety disorder.

**Table 2:** Psychiatric diagnoses of patients receiving IM ketamine therapy

Total *N* = 452	N (%)
Major depressive disorder	420 (93%)
Generalized anxiety disorder	243 (54%)
Post-traumatic stress disorder	126 (28%)
Attention-deficit hyperactivity disorder	107 (24%)
Insomnia	90 (20%)
Anxiety disorder unspecified	54 (12%)
Bipolar disorder	41 (9%)
Panic disorder	38 (8%)
Other mental disorder^a^	35 (8%)
Substance use disorder	23 (5%)
Obsessive compulsive disorder	22 (5%)
Personality disorder	18 (4%)
Eating disorder	15 (3%)
Unspecified mood disorder	14 (3%)
Phobic anxiety disorder	13 (3%)

^**a**^Other mental disorder includes the following diagnoses: dysthymic disorder (*n* = 8), adjustment disorder (*n* = 6), psychotic disorder (*n* = 6), impulse disorder (*n* = 4), acute stress reaction (*n* = 3), delusional disorder (*n* = 3), autism spectrum disorder (*n* = 3), somatoform disorder (*n* = 3), cyclothymic disorder (*n* = 2), and dissociative disorder (*n* = 1)

At baseline, patients were taking a median of 2 (IQR 1–5) psychiatric medications other than ketamine, and 354 (78%) patients were taking at least one psychiatric medication. The most common medication class was antidepressants, and these patients were taking a median of 2 (IQR 1–2) different antidepressants each (Table 3).

**Table 3:** Psychiatric medications at baseline for patients receiving IM ketamine therapy

Total *N* = 452	N (%) of patients	Median (IQR) prescriptions per patient
All psychiatric medications	354 (78%)	3 (2–5)
Antidepressant	294 (65%)	2 (1–2)
Stimulant	142 (31%)	1 (1–1)
Benzodiazepine	141 (31%)	1 (1–1)
Antipsychotic	135 (30%)	1 (1–1)
Anticonvulsant	89 (20%)	1 (1–1)
Sedative/hypnotic	76 (17%)	1 (1–1)
Anticonvulsant/mood stabilizer	57 (13%)	1 (1–1)
Azapirone	36 (8%)	1 (1–1)
Mood stabilizer	22 (5%)	1 (1–1)
Dopamine agonist	17 (4%)	1 (1–1)
Tricyclic antidepressant	9 (2%)	1 (1–1)
Alcohol antagonist	5 (1%)	1 (1–1)
Cannabis/cannabinoid	4 (1%)	1 (1–1)
NMDA receptor antagonist	2 (0.4%)	1 (1–1)
Opioid antagonist	2 (0.4%)	1 (1–1)

### IM ketamine treatment patterns

**Fig. 1:**
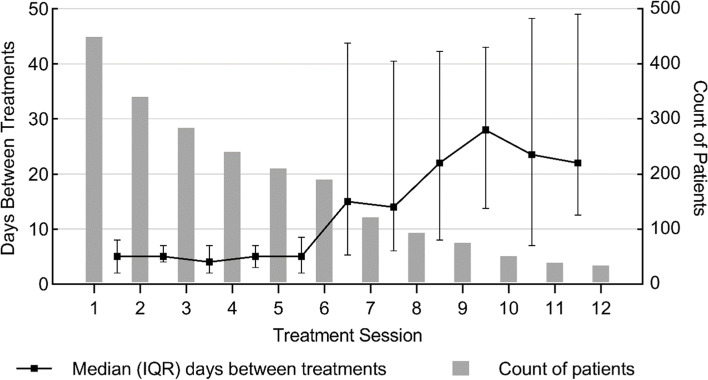
Time between successive IM ketamine treatment sessions 1–12

The number of ketamine treatments patients received ranged from 1 to 48, with a median of 4 (IQR 2–7) treatments. Fig. 1 depicts the frequency of patients’ IM ketamine treatments throughout the typical treatment course.

**Fig. 2:**
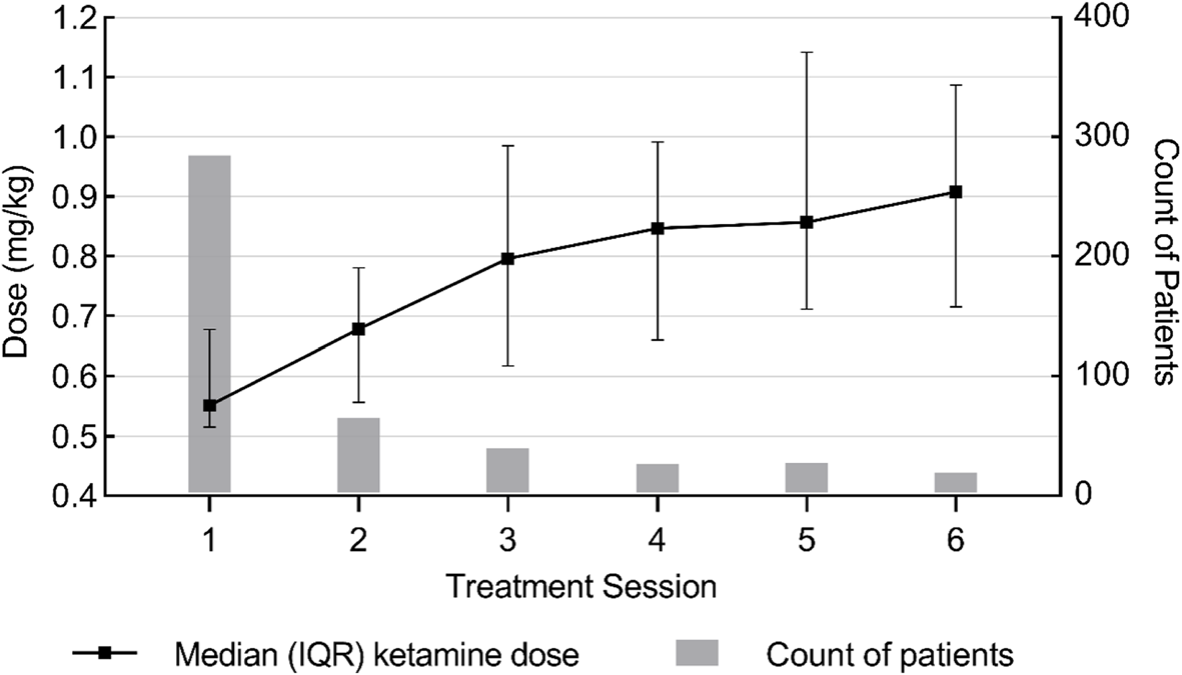
IM Ketamine doses (mg/kg) throughout treatment sessions 1–6

Ketamine dose was positively correlated with patient weight (r(562) = 0.402, *p* < .001). Doses ranged from 0.3 mg/kg to 2.15 mg/kg. Patients were started at a median dose of 0.55 mg/kg (IQR 0.52–0.68) at their first treatment, and this median dose increased to 0.91 mg/kg (IQR 0.72–1.09) at treatment six (Fig. 2). The median dose for maintenance treatments, or treatments 7 to 48 (*N* = 54 treatments), was 1.2 (IQR 0.66–1.45) mg/kg.

### Depression, suicidal ideation, and anxiety outcomes

There were significant reductions in patients’ depression (PHQ-9) and anxiety (GAD-7) scores from baseline to last ketamine treatment (Table 4). Median PHQ-9 scores decreased 38% from 16.0 (IQR 11.3–21.8) or moderately severe depression at baseline, to 10.0 (IQR 6.0–15.0) or moderate depression at last treatment (*p* < .001). Median GAD-7 scores decreased 50% from 14.0 (IQR 8.0–17.0) or moderately severe anxiety at baseline, to 7.0 (IQR 4.3–11.8) or moderate anxiety at last treatment (*p* < .001). The percent of patients reporting any SI, defined as an SI score greater than zero, decreased from 60% (80/133) at baseline to 47% (63/133) at last treatment.

**Table 4:** Depression (PHQ-9) and anxiety (GAD-7) scores by number of IM ketamine treatments received

	*N*^a^	Score at Baseline Median (IQR)	Score at Last Treatment Median (IQR)	*p*-value^b^
Depression – PHQ-9				
All treatment lengths	112	16.0 (11.3–21.8)	10.0 (6.0–15.0)	**<.001**
Patients with 1 treatment	66	13.5 (8.0–21.0)	N/A	N/A
Patients with 2–4 treatments	30	15.0 (7.8–21.0)	12.0 (5.8–15.0)	**<.001**
Patients with 5–6 treatments	38	17.0 (12.5–22.5)	9.0 (6.0–13.0)	**<.001**
Patients with 7–10 treatments	29	15.0 (11.5–22.0)	10.0 (7.0–15.0)	**.001**
Patients with 11–48 treatments	15	20.0 (15.0–24.0)	11.0 (6.0–13.0)	**<.001**
Anxiety – GAD-7				
All treatment lengths	80	14.0 (8.0–17.0)	7.0 (4.3–11.8)	**<.001**
Patients with 1 treatment	50	10.5 (6.0–16.0)	N/A	N/A
Patients with 2–4 treatments	24	10.0 (7.0–14.8)	8.0 (7.8–13.8)	.061
Patients with 5–6 treatments	26	15.5 (11.0–17.0)	7.0 (4.0–11.3)	**<.001**
Patients with 7–10 treatments	20	10.5 (6.0–18.3)	7.5 (5.0–9.8)	**.006**
Patients with 11–48 treatments	10	17.5 (15.5–18.3)	8.5 (5.3–11.0)	**.003**

^a^Only patients with an IM ketamine treatment duration greater than 2 weeks and survey scores available at first and last IM ketamine treatment were included; ^b^Wilcoxon signed-rank tests were conducted; bold lettering = significant change based on a Bonferroni adjusted α of .01

The change in patients’ PHQ-9, SI, and GAD-7 scores from baseline to last treatment negatively correlated with the number of ketamine treatments they had received (Table 5). Thus, additional treatments were correlated with a larger decrease in depression, SI, and anxiety symptom severity.

**Table 5:** Correlations between the number of ketamine treatments patients received and their survey scores

	PHQ-9			SI			GAD-7		
	df	r_s_	*p*-value	df	r_s_	*p*-value	df	r_s_	*p*-value
Correlation between number of treatments and change in survey score^a^	131	−.29	**.0003**	131	−.19	**.014**	90	−.38	**<.0001**

^a^1-tailed Spearman’s correlation; df = degrees of freedom; r_s_ = Spearman’s rank correlation coefficient; bold lettering **=** significant correlation

**Fig. 3:**
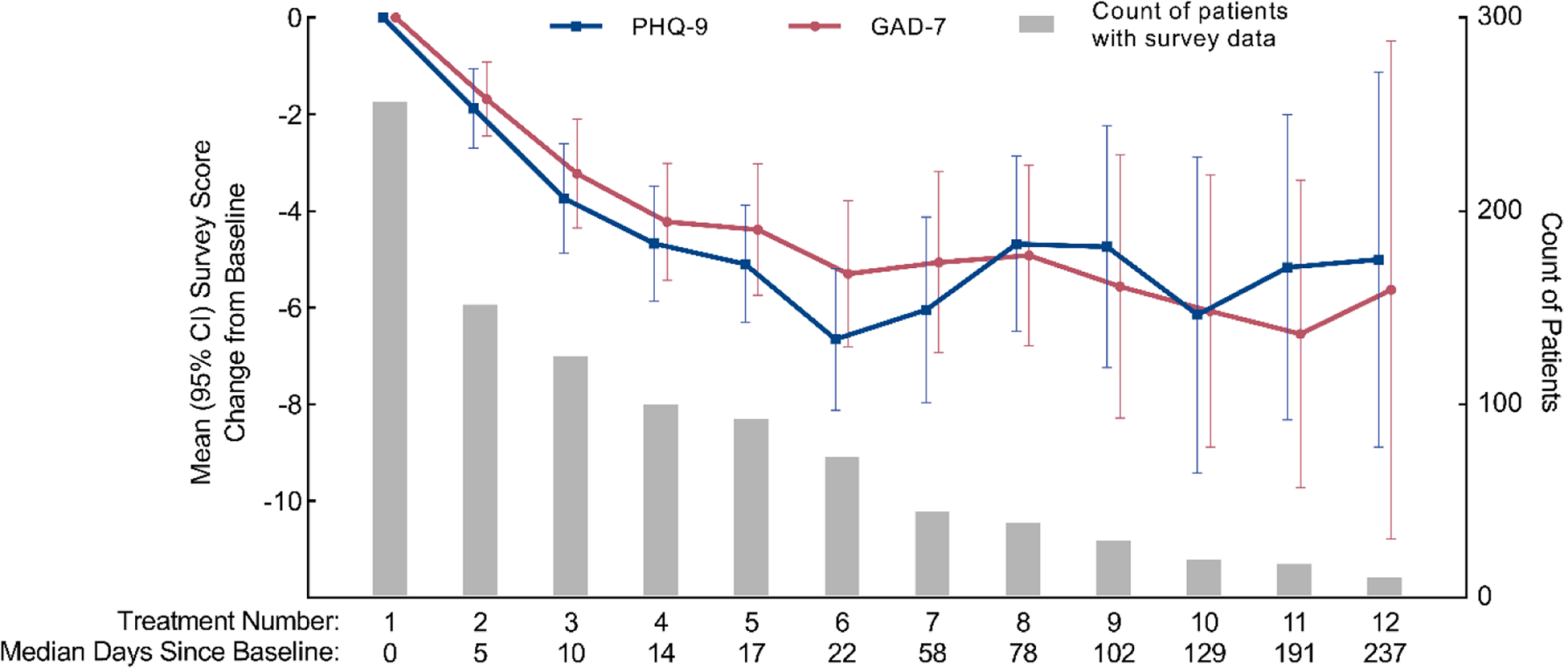
Change in depression and anxiety scores throughout IM ketamine treatments 1–12

Fig. 3 illustrates the change in patients’ depression and anxiety symptoms throughout their treatment course. By treatment 6, PHQ-9 and GAD-7 scores had improved by a mean of 6.6 (SD 6.3) points and 5.3 (SD 5.3) points respectively. We end reporting at treatment 12 due to the sample size decreasing to 12 patients by this treatment. From treatment 6 to treatment 12, mean reductions in PHQ-9 and GAD-7 scores were maintained to be at least 4.7 (SD 5.7) and 4.9 (SD 4.5) points below baseline respectively.

### Safety

Reported AEs during IM ketamine treatment included nausea, vomiting, abnormal vital signs, panic attacks, hallucinations, confusion, potentially unsafe movement, and bladder pain. There were no severe AEs observed. Out of 2532 treatments, an AE occurred during 59 (2.3%) treatments, and all AEs resolved prior to patients leaving the clinic.

Nausea and vomiting were the most common AEs recorded during ketamine treatments. Out of 2532 ketamine treatments administered, 34 (7.5%) patients experienced nausea at 47 (1.9%) treatments, and 20 (4.4%) patients vomited at 26 (1.0%) treatments. Patients were commonly administered 8 mg oral ondansetron prior to their ketamine injection to help prevent nausea and vomiting.

Patients experienced an abnormally large change in vital signs, as noted by the clinician, at 4 (0.16%) out of 2532 treatments. For these 4 treatments, the patient’s average blood pressure elevation from baseline to peak was 28.8 (systolic) and 16.5 (diastolic) mmHg, and the highest blood pressure elevation was 46 (systolic) and 30 (diastolic) mmHg. To help stabilize elevated blood pressure, two patients were administered beta-blockers. Of the four patients who had abnormal vital signs, one patient had a heart rate elevation of 10 bpm. Vital signs stabilized within 30 minutes for all four patients.

Out of 2532 total treatments, three patients experienced a panic attack during 4 (0.16%) treatments. Two out of three of these patients had a history of panic attacks before the AE. Hallucinations occurred at 4 (0.16%) treatments, with confusion at 3 (0.12%) treatments. Also, three patients displayed potentially unsafe movement, including a fall, knocking over equipment, or hyperactivity, at 3 (0.12%) treatments. Finally, one patient reported experiencing bladder spasms and pain during 1 (0.04%) treatment, which resolved after a few minutes.

## Discussion

In this study, we describe the clinical characteristics, treatment patterns, depression, SI and anxiety outcomes, and AEs of patients who received IM ketamine treatment, to help inform the future research and administration of this treatment.

### Population

Patients receiving ketamine treatment were less racially and ethnically diverse than the greater population treated by the state’s mental health authority, for which 2018 data shows that 13.4% of persons were non-white and 15.8% were Hispanic or Latinx (compared to 3 and 4% in this cohort) [36]. One possible explanation for this discrepancy is that the cost of racemic ketamine, which is currently rarely covered by insurance, may limit access for racial/ethnic groups with lower average income [37]. This disproportion could also be due to the relative hesitancy towards trying new prescription drugs amongst minority patients [38]. Research on possible racial/ethnic disparities in ketamine treatment and other novel psychopharmacotherapies is warranted.

At baseline, patients in this cohort presented with a substantial 47% prevalence of lifetime history of physical or sexual abuse, which is comparable to the 56% of psychiatric outpatients and 42% of nonpsychiatric outpatients with a history of abuse reported in 2000 [39], a point in time when abuse was more prevalent [40]. The substantial prevalence of abuse in this cohort mirrors the significant adverse childhood experiences (ACE) scores reported in ketamine-assisted psychotherapy patients by Dore et al. (mean ACE score = 3.6) [18]. Research on the experience and impact of ketamine treatment for patients with a history of trauma is warranted in order to better inform the development of future ketamine treatment protocols for the psychiatric outpatient population.

Patients in this observational study were also clinically complex, in that they had an average of 2.8 psychiatric comorbidities each. This contrasts with cohorts in prospective trials of ketamine therapy where patients with multiple psychiatric comorbidities are either excluded or are rare [2, 6, 41]. In real-world clinical practice, IM ketamine treatment is being utilized to treat a clinically complex population. Importantly, IM ketamine was also used to treat patients with a diverse range of mental illnesses not limited to depression. Since 93% of patients carried a diagnosis of MDD, if follows that at least 7% of patients were receiving ketamine to specifically treat a mental illness other than unipolar depression. Future research on the safety, efficacy and treatment protocols of IM ketamine treatment should be conducted for a variety of psychiatric indications not limited to MDD.

### Treatment patterns

The treatment schedule of patients in this cohort was consistent with protocols from previous trials [20, 21, 42] and case reports [43] of ketamine therapy for treatment-resistant depression, where patients received an acute phase of up to 6 treatments within the span of 1 month, with some patients then receiving less frequent maintenance treatments during the subsequent months or years as determined on a case-by-case basis.

The ketamine dosing schedules of patients at this clinic were collaboratively determined between patients and their care teams. The trend of starting patients at a lower median dose of 0.55 mg/kg and increasing their dose throughout treatment allowed clinicians to determine patients’ individualized sensitivity to ketamine, to provide the greatest possible symptom alleviation while limiting patient’s risk of AEs. The ketamine doses reported in this study are higher than the initial pilot trials reported for psychiatric IM ketamine, for which the highest dose was 0.5 mg/kg [23, 44]. However, the median doses of 0.55 and 0.91 mg/kg at first and sixth ketamine treatments in this cohort are similar to the real-world IM ketamine doses reported by Bonnett et al. for MDD patients [24].

### Depression, suicidal ideation and anxiety outcomes

There were significant improvements in depression and anxiety symptoms from baseline to last ketamine treatment in this cohort. The magnitude of improvements in depression and anxiety were similar to a study which reported on a real-world cohort of patients with a variety of mental illnesses receiving IM and SL ketamine-assisted psychotherapy at an outpatient clinic [18]. Based on a validated conversion of their reported Beck Depression Inventory scores to PHQ-9 scores [45], the average estimated 31% reduction in depression symptoms reported from IM and SL ketamine-assisted psychotherapy was similar to this IM ketamine cohort’s 38% reduction in depression symptoms. Furthermore, the improvements in depression and anxiety symptoms observed among patients in this study are similar to a case series of real-world data from forty MDD patients. In that study, patients received a series of six IM ketamine treatments, and experienced an average reduction in depression and anxiety symptoms of 55% (PHQ-9) and 51% (GAD-7) respectively [24], which is comparable to the 47 and 55% reductions in median PHQ-9 and GAD-7 scores observed among patients in this cohort who received either five or six IM ketamine treatments. The current study shows that, for outpatients with a variety of mental illnesses who receive IM ketamine and other concomitant psychiatric treatments, improvements in average depression and anxiety symptoms occur from before to after IM ketamine treatment.

Few studies have reported on the efficacy of maintenance ketamine treatments, and the current study provides the first cohort-level analysis on maintenance IM ketamine treatments. Other open-label case series’ have reported modest efficacy of twice-weekly maintenance IV ketamine infusions for patients with treatment-resistant depression over 11 months [46, 47], and strong efficacy of weekly maintenance SC ketamine for treatment-refractory GAD or SAD patients over 3 months [48]. For patients in this study who received maintenance IM ketamine treatments, average improvements in depression and anxiety scores of at least 4.7 and 4.9 points were maintained for over 7 months. This long-term maintenance of average symptom improvements suggests possible real-world effectiveness of maintenance IM ketamine treatments for a heterogeneous outpatient psychiatric population. Importantly, as described by Andrade et al., maintenance ketamine may not be required for all patients, and could depend on the initial indication for ketamine [4]. Given the potential clinical benefits of long-term IM ketamine treatment, further research on the long-term effectiveness of ketamine treatment with or without maintenance treatments is warranted for a variety of psychiatric patient populations.

This cohort had a substantial burden of history of suicidality, and our data suggest that levels of suicidal ideation, and possibly suicide risk, may be reduced for this clinically complex patient population upon initiation of IM ketamine treatment. At baseline, the cohort showed a 37% lifetime history of suicide attempts, a burden that is equal to a 2007 characterization study of outpatient psychiatric patients [40]. The percent of patients who reported any suicidal ideation at baseline was 60% (80/133), and this decreased to 47% (63/133) at last ketamine treatment. This could represent a substantial decrease in cohort-level suicide risk, as it has been shown that outpatients who report any suicidal ideation on the SI item of the PHQ-9 are 3-to-7 times more likely to die by suicide in the next 30 days [35]. While our data is promising, future studies on the impact of real-world IM ketamine treatment on not only suicidal ideation, but also suicidal behaviour, is warranted [5].

### Safety

This cohort had a good safety profile, and overall rates of AEs as well as rates of specific AEs were comparable to those reported in previous ketamine studies. AEs occurred during 2.3% of the 2532 treatments in this cohort, which is comparable to the 1.95% of IV ketamine infusions which were discontinued due to AEs as reported in a 2015 systematic review and meta-analysis [49]. The 7.5 and 4.4% of patients who experienced nausea and vomiting, respectively, in this cohort was comparable to the 13 and 6% reported for patients receiving IM and SL ketamine who also received preventative administration of antiemetic medication [18]. Also, unstable vital signs were recorded at 0.16% of treatments, which is lower than the 1% (2/205) of IV ketamine infusions where patients experienced elevated blood pressure as reported by Wan et al. [49]. Prospective studies are warranted which evaluate the follow-up safety of IM ketamine months to years after patients have completed their series of ketamine treatments [50], given the potential widespread application of IM ketamine in community psychiatric and primary care clinics.

## Limitations

Firstly, the generalizability of the patient characteristics and IM ketamine treatment patterns reported here is limited - while this study described the IM ketamine treatment offered at only one private psychiatric clinic network in the US, there is likely heterogeneity in the demographic and clinical characteristics of patients receiving IM ketamine treatment, and the IM ketamine treatment protocols used, at outpatient clinics across North America.

Secondly, ascertainment bias may be present in this study since many variables, namely social, mental health and substance use history variables, and PHQ-9 and GAD-7 scores only had complete data available for a portion of the cohort. For these variables, the subset of patients for whom data was collected may have been influenced by clinicians’ a priori knowledge of each patient’s history and clinical issues. For example, baseline anxiety scores may be overestimated in this study. This is because if a patient had high baseline anxiety, alleviating anxiety may have been an objective for their treatment, thus their clinician may have been more likely to collect GAD-7 scores to track improvement in anxiety over time.

Finally, the results we report regarding the change in patients’ depression, suicidal ideation and anxiety symptoms must be interpreted cautiously. The fact that this study had no control group limits the ability to make causal links between IM ketamine treatment and symptom improvement. Furthermore, patients received open-label treatment, thus the possible placebo effect of ketamine treatment was not evaluated. Nonetheless, the clinical data of this analysis describe significant and long-lasting improvements in depression and anxiety symptoms upon the initiation of IM ketamine treatment.

## Conclusions

IM ketamine is being utilized as an off-label treatment for psychiatric outpatients with a variety of mental illnesses and multiple psychiatric comorbidities. On average, psychiatric outpatients experienced a reduction in depression and anxiety symptoms from before to after a course of IM ketamine treatment, and average improvements in depression and anxiety symptoms did not regress to baseline during the maintenance treatment phase. There were no major safety concerns during IM ketamine treatment. Prospective studies are required to confirm the long-term safety and effectiveness of psychiatric IM ketamine treatment.

## Supplementary Information

**Additional file 1: Supplemental Table 1.** Baseline medical and substance use history of patients receiving IM ketamine therapy (self-reported).

**Additional file 2: Supplemental Table 2.** Family mental health history of patients receiving IM ketamine therapy (self-reported).

## Data Availability

The datasets generated and/or analysed during the current study are available from the corresponding author, SA, on reasonable request, but are not publicly available due to patient privacy restrictions.

## Abbreviations

ACEs: Adverse childhood experiences
AE: Adverse event
DSM-V: Diagnostic and Statistical Manual of Mental Disorders, 5th Edition
EHR: Electronic health records
GAD: Generalized anxiety disorder
GAD-7: Generalized Anxiety Disorder-7
IM: Intramuscular
IN: Intranasal
IQR: Interquartile range
IV: Intravenous
MDD: Major depressive disorder
PHQ-9: Patient Health Questionnaire
PTSD: Post-traumatic stress disorder
SAD: Social anxiety disorder
SD: Standard deviation
SI: Suicidal ideation
SL: Sublingual
