# Supplementary material for: Real-world depression, anxiety and safety outcomes of intramuscular ketamine treatment: a retrospective descriptive cohort study
Source: BMC Psychiatry. 2022 Oct 3;22:634. doi: 10.1186/s12888-022-04268-5 (PMC9528178; doi:10.1186/s12888-022-04268-5)
Supplement: Supplementary file 1 — Additional file 1: Supplemental Table 1. Baseline medical and substance use history of patients receiving IM ketamine therapy (self-reported). [file 12888_2022_4268_MOESM1_ESM.pdf]

Supplemental Table 1 Baseline medical and substance use history of patients receiving IM ketamine therapy (self-reported)

	No. of patients with available data	N (%)
**Medical History – Physical Conditions**		
Acute or chronic pain	146	47 (32.2%)
Asthma	152	36 (23.7%)
GI issues	120	34 (28.3%)
High blood pressure	126	26 (20.6%)
Thyroid disease	139	26 (18.7%)
GERD/Reflux	133	24 (18.0%)
Sleep apnea	125	23 (18.4%)
Fibromyalgia	126	16 (12.7%)
Muscle/bone/joint disorders	113	14 (12.4%)
Gynecologic issues	109	13 (11.9%)
Neurological conditions	110	12 (10.9%)
TBI	117	12 (10.3%)
Endocrine issues	109	10 (9.2%)
Seizures/epilepsy	126	9 (7.1%)
Cancer	120	7 (5.8%)
Diabetes	132	7 (5.3%)
Cardiovascular issues	109	6 (5.5%)
Respiratory issues	112	6 (5.4%)
COPD/emphysema/bronchitis	120	4 (3.3%)
Kidney problems	107	4 (3.7%)
Liver problems	104	3 (2.9%)
Hematological issues	101	2 (2.0%)
Infectious diseases	104	2 (1.9%)
Stroke/TIA	121	1 (0.8%)
**Substance Use History**		
Alcohol consumption	250	
Denies history of use		109 (43.6%)
Occasional		98 (39.2%)
Moderate		37 (14.8%)
Heavy		6 (2.4%)
Alcohol-related blackouts, delirium tremens or seizures	227	21 (9.3%)
Smoking history	281	
Never		209 (74.4%)
Formerly		40 (14.2%)
Currently – some days		13 (4.6%)
Currently – every day		19 (6.8%)
Benzodiazepine use	227	

Denies history of use		183 (80.6%)
Previous use – denies current use		13 (5.7%)
Currently uses		31 (13.7%)
Cannabis use	224	
Denies history of use		93 (41.5%)
Previous use – denies current use		75 (33.5%)
Recreational use		43 (19.2%)
Prescribed medicinal cannabis		13 (5.8%)
Cocaine or amphetamine use	234	
Denies history of use		193 (82.5%)
Previous use – denies current use		36 (15.4%)
Currently Uses		5 (2.1%)
Opiate (pain pills/heroin) use	233	
Denies history of use		186 (79.8%)
Previous use – denies current use		37 (15.9%)
Currently Uses		10 (4.3%)
Prior psychedelic drug use	225	46 (20.4%)
