# Supplementary material for: Real-world depression, anxiety and safety outcomes of intramuscular ketamine treatment: a retrospective descriptive cohort study
Source: BMC Psychiatry. 2022 Oct 3;22:634. doi: 10.1186/s12888-022-04268-5 (PMC9528178; doi:10.1186/s12888-022-04268-5)
Supplement: Supplementary file 2 — Additional file 2: Supplemental Table 2. Family mental health history of patients receiving IM ketamine therapy (self-reported). [file 12888_2022_4268_MOESM2_ESM.pdf]

Supplemental Table 2 Family mental health history of patients receiving IM ketamine therapy (self-reported)

Total *N* = 266	N (%) of patients with affected family member
Family history of mental illness	236 (88.7%)
Family history of attempted suicide	37 (13.9%)
Family member died by suicide	2 (0.8%)
Family history of depression	165 (62.0%)
Family history of anxiety disorder	132 (49.6%)
Family history of substance use disorder	92 (34.6%)
Family history of attention-deficit hyperactivity disorder	63 (23.7%)
Family history of other mood disorder	61 (22.9%)
Family history of post-traumatic stress disorder	35 (13.2%)
Family history of obsessive-compulsive disorder	32 (12.0%)
Family history of personality disorder	20 (7.4%)
Family history of schizophrenia/schizoaffective disorder	13 (4.9%)
